# Legionella‐induced dysarthria and rhabdomyolysis with acute renal failure achieving recovery

**DOI:** 10.1002/ccr3.8628

**Published:** 2024-03-07

**Authors:** Husam El Sharu, Soban Ahmad, Hunter Coore

**Affiliations:** ^1^ Internal Medicine East Carolina University Health Medical Center Greenville North Carolina USA

**Keywords:** corpus callosum lesion, dysarthria, hemodialysis, Legionella, rhabdomyolysis

## Abstract

**Key Clinical Message:**

Legionnaires' disease, often presenting as pneumonia, can uncommonly manifest with extrapulmonary symptoms such as cerebellar involvement and rhabdomyolysis. This case emphasizes the successful resolution of dysarthria and renal dysfunction with prompt Legionella treatment, underscoring the importance of vigilance for diverse manifestations in Legionella infections.

**Abstract:**

Legionnaires' disease usually presents with pneumonia and a few extrapulmonary manifestations, such as neurological, musculoskeletal, and cutaneous manifestations. However, cerebellar involvement and rhabdomyolysis as an association with Legionella are not frequently encountered. We present a case of Legionella‐induced rhabdomyolysis requiring hemodialysis and dysarthria that resolved with Legionella treatment.

## Graphical abstract

Brain MRI showing enlarged, edematous splenum of the corpus callosum along a well‐defined ovoid lesion in the midline of the splenium of the corpus callosum consistent with a cytotoxic lesion of the corpus callosum as a result of *legionella pneumophila*.

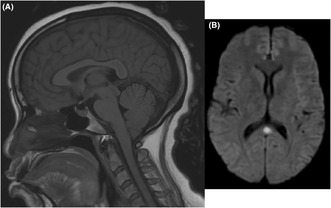

## 1 INTRODUCTION

Legionellaceae comprises a single genus, Legionella, which is a facultatively intracellular, obligatory aerobic, gram‐negative bacilli and nutritionally fastidious, requiring l‐cysteine and ferric salts. It is transmitted through water particles and may cause hospital or community‐acquired pneumonia. It may cause two distinct spectrums of disease: Pontiac fever and Legionnaires disease (LD), which is 90% caused by *Legionella pneumophila*. While Pontiac fever is a self‐limited, febrile, benign, non‐pneumonic disease associated with exposure to Legionella, LD patients usually present with pneumonia symptoms such as fever and cough along with evidence of consolidation on chest x‐ray (CXR). LD can also present with extrapulmonary manifestations, including neurological, musculoskeletal, and cutaneous manifestations.^1^ Neurological manifestations may include transient encephalopathy and focal deficits. Nevertheless, cerebellar involvement and cytotoxic corpus callosum lesions (CCCL) as complications of Legionella are not routinely described in the literature.^2^ Also, the association between Legionella infection and rhabdomyolysis leading to acute kidney injury (AKI) is not commonly linked to practicing physicians.^3^, ^4^ Herein, we present a case of Legionnaires' disease that presented with dysarthria and rhabdomyolysis leading to anuric AKI requiring hemodialysis, both of which resolved with the treatment of LD.

## 2 CASE PRESENTATION

A 44‐year‐old African American female presented to the hospital with a 2‐day duration of dry cough, myalgias, and fever of 102.1°F. She was at a social event a few days before symptoms onset. The remainder of the review of systems was unremarkable. Medical history was significant for well‐controlled essential hypertension, hyperlipidemia, type 2 diabetes mellitus, and tobacco use. Her home medications include Amlodipine, Atorvastatin, Hydrochlorothiazide, and metformin. A physical exam revealed bronchial breath sounds over the left lower lobe with rales. She had mild dysarthria without any facial deviation. The rest of the physical exam was unremarkable. Initial laboratory results at presentation were significant for marked leukocytosis, hyponatremia, and elevated serum creatinine (Table 1). Given AKI, anuria, and diffuse myalgias, serum creatinine phosphokinase (CPK) levels were checked and were 11,275 U/L.

**TABLE 1:** Laboratory results at presentation and at 2 weeks post‐discharge (after she finished hemodialysis).

Parameter	Results at presentation	Results at 2 weeks after discharge	Normal range
Hemoglobin (mg/dL)	13.4	9.3	12.0–16.0
White blood cells (1000/μL)	17.4	9.5	4.0–12.0
Sodium (mEq/L)	128	141	136–145
Bicarbonate (mmol/L)	18	19	21–32
Anion gap (mEq/L)	24	10	4–12
Potassium (mEq/L)	3.3	4.2	3.5–5.1
Creatinine (mg/dL)	15.22	1.28	0.50–1.33
Blood urea nitrogen (mg/dL)	119	51	7–18
Creatinine phosphokinase (CPK) (U/L)	11,275	Not available	29–168
Thyroid‐stimulating hormone (uIU/mL)	1.34	Not available	0.35–4.94
sCorrected calcium (mg/dL)	9.4	9.8	8.4–10.1
Alkaline phosphatase (U/L)	99	117	40–150
Aspartate transaminase (U/L)	193	25	10–37
Alanine transaminase (U/L)	57	31	12–78
Total bilirubin (mg/dL)	0.8	<0.2	0.2–1.5
Albumin (g/dL)	2.8	4.2	3.4–5.0

## 3 DIFFERENTIAL DIAGNOSIS AND INVESTIGATION

Initial differential diagnoses include pneumonia in the setting of fever, cough, and myalgias. Pneumonia in the setting of hyponatremia would possibly suggest Legionella pneumonia(LP). Additionally, given the neurological symptoms, a suspicion of concomitant stroke was also on the differential. Accordingly, urine Legionella antigen was tested and was found to be positive. Additional workups, including human immunodeficiency virus, respiratory viruses, and blood cultures, were unremarkable. Antinuclear antibody was positive with a titer of 1:320, but Anti‐double‐stranded DNA, antiphospholipid, scleroderma antibodies, and complements levels were unremarkable. Serum and urine protein electrophoresis showed evidence of non‐nephrotic range proteinuria without monoclonal proteins.

**FIGURE 1:**
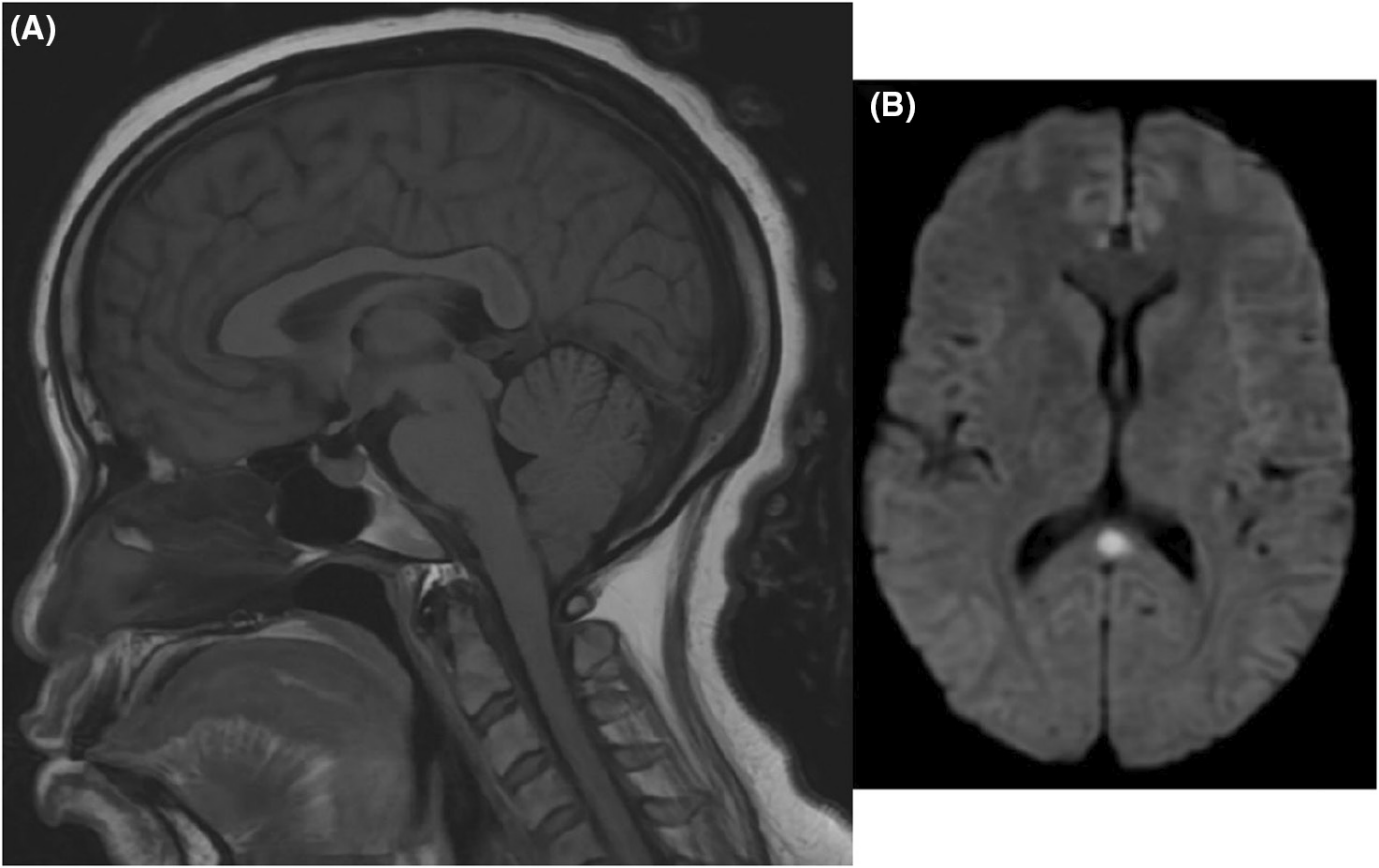
(A) Sagittal TI FLAIR reveals an enlarged and edematous corpus callosum, including the splenum. (B) Axial diffusion‐weighted imaging reveals a well‐defined ovoid lesion in the midline of the splenium of the corpus callosum with associated restricted diffusion.

CXR showed a small left pleural effusion with adjacent left lower consolidation. Given neurologic findings, computed tomography of the head was performed that was unremarkable. Magnetic resonance imaging (MRI) of the brain showed a cytotoxic ovoid lesion in the splenium of the corpus callosum (Figure 1 A,B). Retroperitoneal ultrasound showed normal‐sized kidneys with no hydronephrosis.

The patient received only 1‐time dose of broad‐spectrum antibiotics, including vancomycin and piperacillin‐tazobactam, and was quickly de‐escalated to azithromycin for treating LD for a total of 14 days of antibiotics. With intravenous (IV) hydration and appropriate antibiotic therapy, her CPK levels successfully trended down. However, her urine output, serum creatinine, and blood urea nitrogen failed to improve. Due to deteriorating azotemia, anuria, and initial signs of uremia such as nausea, altered taste, and reduced appetite, hemodialysis was initiated. It was sustained as an outpatient measure for a limited period of 2 weeks as a transitional approach, and subsequently, she successfully discontinued dialysis.

### 3.1 Outcomes and follow‐up

After completing a 2‐week course of antibiotic therapy, the patient experienced a resolution of dysarthria and notable improvements in urinary output, sodium level, and renal function. During her 2‐week follow‐up, the patient's serum creatinine level measured 1.28 mg/dL, the estimated glomerular filtration rate (eGFR) was 53 mL/Min/1.73 m^2^, and the creatinine clearance was 56 mL/Min. At the eight‐week follow‐up, further improvement was observed, with a serum creatinine level decreasing to 1.04 mg/dL and an eGFR of 64 mL/Min/1.73 m^2^.

## 4 DISCUSSION

The most common and virulent strain is *Legionella pneumophila*, particularly Lp1. Among many virulence factors, the key virulence factor is the ability to evade endocytic fusion with lysosomes and subsequent replication within alveolar macrophages and other cell organelles using the Dot (defect in organelle trafficking)/Icm (intracellular multiplication) type IV secretion system—An essential protein translocation system plays a crucial role in the pathogen's intracellular survival and replication referred to as the Legionella‐containing vacuole.^5^

LP accounts for 2%–9% of all community‐acquired pneumonia cases. However, LD is under‐recognized and under‐reported, with reported cases representing 5% of actual cases.^6^ Risk factors include chronic lung disease, age >50, smoking, and use of steroids.^6^ Aside from pneumonia, LD can manifest with extrapulmonary symptoms. Raising awareness about uncommon presentations such as rhabdomyolysis and dysarthria is crucial, as delayed treatment may worsen mortality.^7^ LD diagnosis is typically made through urine antigen test, but 5% of cases may require culture. Treatment is mainly with respiratory fluoroquinolones or macrolides. Extrapulmonary manifestations are usually treated supportively.^6^

Neurological manifestations commonly associated with LD involve meningitis, encephalitis, and brain stem abnormalities. While cerebellar involvement is rarely described as a result of LD, dysarthria, and ataxia are the most common presenting symptoms, which are thought to result from LD‐induced neurotoxin or immune reactions.^8^ CCCL is a secondary lesion with a restricted MRI diffusion pattern. It can result from dysfunction of the callosal neurons and microglia due to increased cytokine glutamate levels, especially in the splenum due to higher cytokine concentrations.^9^ It is worth noting that the association between CCCL and LD was described previously.^10^ Thus, finding CCCL in the setting of pre‐existing pneumonia should raise the concern for Legionella testing. In a previous report, symptoms were fully resolved with antibiotics without steroids.^11^

Although rare, pneumonia‐induced rhabdomyolysis, a syndrome characterized by elevated serum CPK causing myoglobinuria and renal dysfunction, is well described in the literature. The primary organisms are *S*. *pneumoniae* and *L*. *pneumophila*.^12^ The mortality of *L*. *pneumophila* pneumonia is 5%–15%. However, when combined with rhabdomyolysis and AKI, the mortality increases to 40%.^13^ Since the description of the first case by Posner et al. in 1980, the relationship between LD and rhabdomyolysis has become more established.^14^ The exact mechanism is not fully understood. A possible theory is possibly direct bacterial invasion or toxin‐mediated renal injury resulting in oliguric AKI. Another proposed mechanism includes Legionella, causing an immune‐mediated reaction toward the kidney.^4^, ^15^ Similar to our patient, most patients with LD‐induced AKI required dialysis, with the most common indications being uremic symptoms and oliguria.^4^, ^13^ It is noteworthy that her home medications, which she has been receiving for a long time, or the 1‐time dose of vancomycin, could be the culprit in her multi‐organ damage.

Interestingly, our patient developed multiorgan dysfunction, which is not a typical presentation of LD managed with antibiotics and hemodialysis with favorable outcomes. Notably, only one case in the literature with a similar presentation to ours involved renal and neurological dysfunction. Unlike our case, the patient was treated with a 14‐day course of azithromycin and reported improvement in dysarthria and ataxia without complete resolution on a one‐month follow‐up.^16^ In a small case series that followed the outcomes of neurological dysfunction of LD patients. It concluded that out of 17 patients, 12 patients had persistent neurological dysfunction.^8^

## 5 CONCLUSION

In conclusion, this case report highlights the unusual presentation of Legionnaires' disease with cerebellar involvement and rhabdomyolysis. Prompt diagnosis, antibiotic therapy, and hemodialysis led to the resolution of symptoms, emphasizing the importance of recognizing atypical manifestations for timely intervention and enhanced patient outcomes in Legionella infections.

## AUTHOR CONTRIBUTIONS

**Husam El Sharu:** Conceptualization; methodology; project administration; writing – original draft; writing – review and editing. **Soban Ahmad:** Conceptualization; investigation; methodology; writing – review and editing. **Hunter Coore:** Supervision; validation; visualization; writing – review and editing.

## FUNDING INFORMATION

None.

## CONFLICT OF INTEREST STATEMENT

None.

### CONSENT

Written informed consent was obtained from the patient to publish this report in accordance with the journal's patient consent policy.

## Data Availability

All generated and analyzed data for this study are included in the manuscript.
